# Spillover of an endemic avian Influenza H6N2 chicken lineage to ostriches and reassortment with clade 2.3.4.4b H5N1 high pathogenicity viruses in chickens

**DOI:** 10.1007/s11259-023-10258-z

**Published:** 2023-11-15

**Authors:** Celia Abolnik

**Affiliations:** Department of Production Animal Studies, Faculty of Veterinary Science, University of Pretoria, Onderstepoort, South Africa

**Keywords:** Avian Influenza virus, H6N2, H5N1, Genome sequencing, Reassortment

## Abstract

Prior to 2017, chicken production in South Africa had only ever been affected by an endemic strain of H6N2 low pathogenic avian influenza (LPAI), but since 2017, an outbreak of Goose/Guangdong clade 2.3.4.4b H5N8 high pathogenicity avian influenza (HPAI) introduced by wild birds, followed by clade 2.3.4.4b H5N1 HPAI (2021-present), affected the country. In the present study, the viruses from seven cases of H6N2 LPAI from commercial poultry between October 2019 and August 2020 were genome-sequenced along with an H5N2 HPAI virus, and phylogenetic analysis was performed. The H5N2 HPAI virus caused localized outbreaks in a small-scale chicken farm and a large commercial layer farm in the KwaZulu-Natal province between late October and early December 2022. The phylogenetic results confirmed the first incidence of the chicken-adapted H6N2 lineage in commercial ostriches in the Western Cape province, with a likely epidemiological origin in chickens from the KwaZulu Natal province. The results also showed that the H5N2 HPAI virus was a novel reassortant of PB2, PB1, PA, NP and NA genome segments derived from a parental H6N2 virus that circulated in region, whereas the HA, M and NS genome segments were derived from sub-genotype SA10 H5N1 HPAI parental virus that had circulated in the local wild bird reservoir since July 2021.

**Supplementary Information:**

The online version contains supplementary material available at 10.1007/s11259-023-10258-z.

## Introduction

Influenza A virus (IAV; family *Orthomyxoviridae*) is a single-stranded negative sense RNA virus with an eight-segmented genome. Wild aquatic birds are the primordial reservoirs of antigenically diverse low pathogenicity avian influenza (LPAI) viruses (Webster et al. 1992), but in recent years, migratory aquatic birds became a reservoir for Goose/Guangdong (Gs/GD) sub-lineages of H5Nx high pathogenicity avian influenza (HPAI) viruses, that since 2006 have spread globally in multiple pandemic waves (Lee et al. 2017). Due to its geographic position at the southernmost tip of the African continent, South Africa remained free of Gs/GD H5Nx HPAI until 2017. In that year, the ecology of the virus changed somehow, and intra-African migratory ducks brought Gs/GD clade 2.3.4.4b H5N8 HPAI viruses further south into the region. The South African poultry industry was nearly decimated during the 2017–2018 H5N8 HPAI outbreaks, and again in 2021–2023 by a second wave caused by clade 2.3.4.4b H5N1 HPAI viruses. Molecular analysis of H5N1 HPAI viruses from 2021 to 2022 revealed fifteen distinct sub-genotypes (SA1 to SA15) in the country, some of which were restricted to certain regions (Abolnik et al. 2019).

Prior to 2017, chicken production in South Africa (SA) had only ever been affected by an endemic strain of H6N2 LPAI. The H6N2 virus emerged in the early 2000’s in the Kwa-Zulu-Natal (KZN) province, and eventually spread to other provinces via the movement of infected chickens. H6N2 causes respiratory signs and drops in egg production, compelling the use of a whole inactivated H6N2 vaccine, but this combined with the sale of live spent hens has contributed to the continued endemic circulation of H6N2 in some provinces for more than two decades now. The H6N2 chicken-adapted virus has not been detected in wild birds over two decades of active surveillance in the country, nor have any of its genome segments reassorted with other wild bird LPAI viruses in the continent (Abolnik et al. 2023).

On the 25th of October 2022, while some regions in SA were still experiencing clade 2.3.4.4b H5N1 outbreaks, a small-scale farmer in the Msunduzi Local municipality of the KZN province reported the deaths of 58 of 200 chickens. Samples were taken by the state veterinarian, and surprisingly, the cause was diagnosed as H5N2 HPAI by a national veterinary laboratory. The following month, H5N2 HPAI was again identified as the cause of increased mortalities in a commercial layer farm near Cato Ridge in the neighboring Mkhambathini Local Municipality. On that farm, a house tested positive on the 29th of November 2022, followed by a second house sampled a week later on the 6th of December 2022. The entire farm was culled to stop the further spread of infection (Department of Agriculture, Land Reform and Rural Development, 2023). The purpose of this study was to perform genetic characterization to determine the epidemiological origin/s of the H5N2 HPAI virus that caused the localized outbreak in KZN in late 2022. The genomes of H6N2 viruses from cases in poultry from 2019 to 2021 were also sequenced as part of this study.

## Methods

Assurecloud Laboratory (Pty) Ltd received clinical samples (tracheal and cloacal swabs or tissues) from their clients and performed all RNA extractions, with IAV detection and subtyping by real-time reverse transcription PCR as described previously (Abolnik et al. 2019). Samples from chickens originated from commercial layer flocks in the Gauteng and KwaZulu-Natal provinces with a suspicion of IAV infection, where respiratory signs, drops in egg production or increased mortalities were noted (Table 1). Tracheal swabs from apparently healthy commercial ostriches in the Western Cape province had been tested as part of routine surveillance. Extracted RNA was forwarded to the University of Pretoria for further analysis. IAV genome amplification reverse transcription PCR, Ion Torrent Sequencing, and genome assembly were performed as described elsewhere (Abolnik et al. 2019, 2023). For convenience, IAV’s eight genome segments’ sequences are referred to as PB2 (segment 1; polymerase B2), PB1 (segment 2; polymerase B1), PA (segment 3; polymerase A plus PA-X), HA (segment 4; hemagglutinin), NP (segment 5; nucleocapsid), NA (segment 5; neuraminidase), M (segment 7; matrix 1 plus matrix 2e) and NS (segment 8; non-structural 1 plus nuclear export protein).

MEGA-X (v10.2.5) (Kumar et al. 2018) was used to concatenate genome segment sequences. Multiple sequence alignments of the eight individual segments and concatenated segments were prepared using the MAFFT v.7 (https://mafft.cbrc.jp/alignment/server/index.html; Katoh and Stadley, 2013) and BioEdit (Hall 1999). Blast homology searches were conducted in the GISAID EpiFlu database (https://platform.epicov.org/). Phylogenies were reconstructed using maximum likelihood (ML) analysis in IQ-Tree v2.0.3 with 1,000 ultrafast bootstrap replicates (Trifinopoulos et al. 2016), and the ML consensus trees were visualized using FigTree 1.4.4 (http://tree.bio.ac.uk/software/figtree/).

The time to the most recent common ancestor (RCA) was inferred from the dated maximum clade credibility (MCC) tree in BEAST v.2 software (Bouckaert et al. 2019). The MCC tree was reconstructed using a Hasegawa–Kishino–Yano nucleotide substitution model with a gamma distribution of substitution rates, a Coalescent Bayesian Skyline model and a Relaxed Lognormal clock. Markov chain Monte Carlo chains of 50 million iterations were performed and assessed with Tracer v1.7.2 (Rambaut et al. 2018) to ensure that an effective sample size of > 200 was achieved, with statistical uncertainty of the nodes reflected in values of the 95% highest posterior density (HPD). The consensus MCC tree with common ancestor heights was summarized in TreeAnnotator v.2.6.6 and visualized using FigTree v.1.4.2. Sequences generated in this study were deposited in the GISAID EpiFlu database under the accession numbers provided in Table 1.

**Table 1:** Viruses sequenced in this study

Strain	Sampling date	Location	Accession number
A/chicken/South Africa/387,087/2019 (H6N2)	21 Oct 2019	Randburg, Gauteng province	EPI_ISL_12864553
A/chicken/South Africa/636,697/2020 (H6N2)	6 May 2020	Camperdown, KwaZulu-Natal province	EPI_ISL_12869978
A/ostrich/South Africa/647,367/2020 (H6N2)	6 Jun 2020	Calitzdorp, Western Cape province	EPI_ISL_12869983^a^
A/ostrich/South Africa/644,344/2020 (H6N2)	17 Jun 2020	Oudtshoorn, Western Cape province	EPI_ISL_12869982 ^a^
A/chicken/South Africa/645,275/2020 (H6N2)	18 Jun 2020	Camperdown, KwaZulu-Natal province	EPI_ISL_12869977
A/chicken/South Africa/654,182/2020 (H6N2)	13 Aug 2020	Camperdown, KwaZulu-Natal province	EPI_ISL_12869980
A/chicken/South Africa/654,160/2020 (H6N2)	13 Aug 2020	Camperdown, KwaZulu-Natal province	EPI_ISL_12869979 ^a^
A/chicken/South Africa/69,103/2022 (H5N2, HPAI)	29 Nov 2022	Cato Ridge, KwaZulu-Natal province	EPI_ISL_17837307

^a^ Partial genomes

## Results and discussion

Seven H6N2 LPAI viruses in commercial poultry between October 2019 and August 2020 were sequenced in this study along with the reassortant H5N2 HPAI virus detected in layers in Cato Ridge, KZN, in November 2022. Phylogenetic analysis determined that the H6N2 viruses contained only H6N2-associated segments belonging to H6N2 sub-lineage I, and clustered according to their geographic location, i.e., a sub-clade comprised of strains from Gauteng and North-West provinces, and a sub-clade for KZN (Supplemental Fig. 1(a); (Abolnik et al. 2019). The H6N2 viruses detected in commercial ostriches in the Western Cape province, A/ostrich/South Africa/647,367/2020 and A/ostrich/South Africa/644,344/2020, are located within the KZN sub-clade, and within this subclade, the ostrich viruses shared an RCA with A/chicken/South Africa/654,160/2020 (this study) and A/chicken/South Africa/chicken/H44954/2016 (Abolnik et al. 2019) in the PB1, PA, NP, NA, M and NS genes. Thus, there is strong phylogenetic evidence that the H6N2 strain that affected ostriches in the Western Cape province in 2020 originated in the KZN province sector, where there are no ostrich farms. Transmission from KZN to the Western Cape province was likely via the transportation of spent hens, with fomite introduction into ostrich farms. Wild birds are not suspected to be reservoirs of the chicken-adapted H6N2 strain, because of the genetic purity of the lineage (a lack of reassortment with wild bird associated IAV genes), and the H6N2 virus has never been detected in wild birds during active surveillance. There were no genetic markers associated with prolonged circulation in ostriches (e.g., PB2 E627K) (Abolnik et al. 2016). This is the first report of a spillover of the chicken-adapted H6N2 lineage to ostriches, more than two decades after it first emerged in South Africa.

**Fig. 1:**
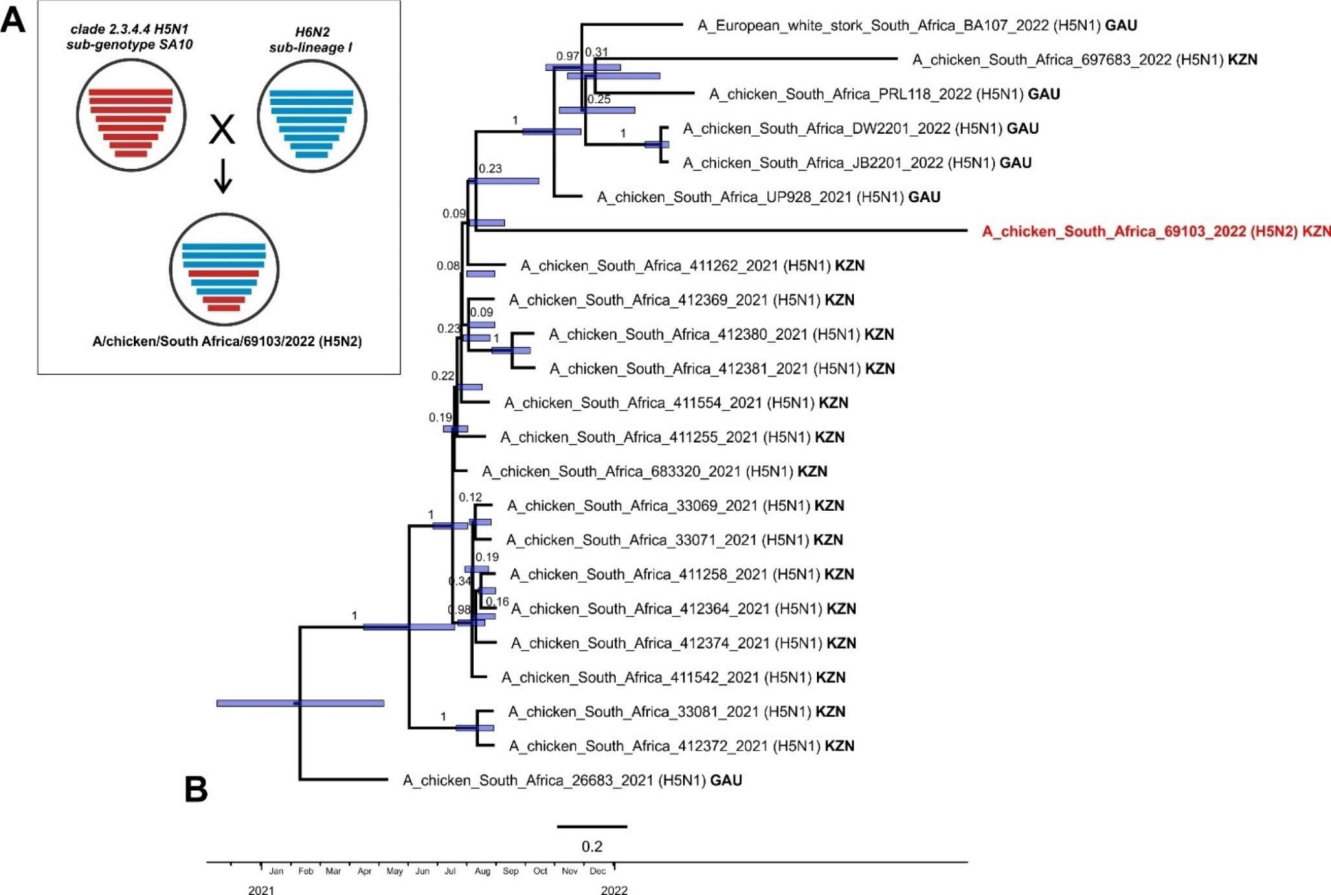
(**A**) Schematic diagram of the origins of the genome segments of the reassortant H5N2 HPAI virus as determined by phylogenetic analysis. (**B**) Time-scaled maximum clade credibility tree of the concatenated HA, M and NS sequences of clade 2.3.4.4b H5N1 HPAI sub-genotype SA10 viruses from South Africa in 2021–2022, rooted with A/chicken/South Africa/26,683/2021 (sub-genotype SA1). The node values represent the posterior probability and the blue bars represent the 95% highest posterior probability range. The H5N2 HPAI reassortant virus is indicated in red. GAU- Gauteng province; KZN- KwaZulu-Natal province

Sequence analysis determined that in the reassortant virus A/chicken/South Africa/69,103/2022 (H5N2, HPAI), the HA, M and NS segments were derived from a clade 2.3.4.4 H5N1 virus (Fig. 1, Supplemental Fig. 1(b), and the PB2, PB1, PA, NP and NA segments were derived from the chicken H6N2 lineage (Supplemental Fig. 1(a). The latter were completely unrelated to any clade 2.3.4.4b H5N1 viruses, with nucleotide sequence identities of < 90%. In the H6N2-origin genes, three KZN viruses sequenced in this study, A/chicken/South Africa/636,697/2020, A/chicken/South Africa/654,182/2020 and A/chicken/South Africa/645,275/2020, isolated from layer hens in the Camperdown region (close to Cato Ridge), share an RCA with the H5N2 reassortant virus (Supplemental Fig. 1(a). To maximize the genetic information, the H5N1-derived HA, M and NS segments were concatenated and aligned with the H5N1 viruses from the South African outbreaks in 2021–2022. The H5N2 reassortant virus clustered within the sub-genotype designated as SA10 (Fig. 1), comprising the strains isolated from the outbreaks from KZN from late June to early September 2021 and in early 2022 in Gauteng (Abolnik et al. 2023). The long branch of A/chicken/South Africa/69,103/2022 (H5N2) and its basal position to the sub-cluster of SA10 viruses indicates that the ancestral H5N1 virus had likely been circulating in the wild bird reservoir in KZN for some time. Although the posterior probability is low, the dating indicates the progenitor was already present in the KZN region in late July 2021 (95% HPD July-October 2021) (Fig. 1), but it was not associated with any outbreak that affected chickens at the time. Furthermore, A/chicken/South Africa/69,103/2022 (H5N2) was unrelated to an H5N1 outbreak in a layer farm in the same Cato Ridge area just a month prior, on 18 September 2022 (Fig. 1).

In conclusion, H6N2 has continued to circulate in chickens in South Africa for more than two decades, but this is the first time that the chicken-adapted strain was detected in ostriches. Ironically, the H6N2 chicken lineage originated in farmed ostriches in the Western Cape province, through a reassortment of H9N2 and H6N8 viruses in the late 1990’s, before spreading to the KZN province with spent hens (Abolnik et al. 2007). The reassortment event with clade 2.3.4.4b H5N1 HPAI in 2022 is only known case involving the H6N2 endemic virus. H5N2 HPAI caused two localized outbreaks in October and November 2022 in a relatively small region of the KZN province, but since the index case wasn’t available for analysis, it was impossible to determine whether the second outbreak, sequenced here, was as the result of a fomite spread from the first outbreak, or whether reassortment events occurred independently. Nonetheless, no further spread of H5N2 HPAI was reported after December 2022.

### Electronic supplementary material

Below is the link to the electronic supplementary material.

Supplementary Material 1

## Data Availability

All sequences generated in this study are available in the GISAID EpiFlu database.
