# Supplementary material for: Spillover of an endemic avian Influenza H6N2 chicken lineage to ostriches and reassortment with clade 2.3.4.4b H5N1 high pathogenicity viruses in chickens
Source: Vet Res Commun. 2023 Nov 15;48(2):1233–7. doi: 10.1007/s11259-023-10258-z (PMC10998810; doi:10.1007/s11259-023-10258-z)
Supplement: Supplementary file 1 — Supplementary Material 1 [file 11259_2023_10258_MOESM1_ESM.docx]

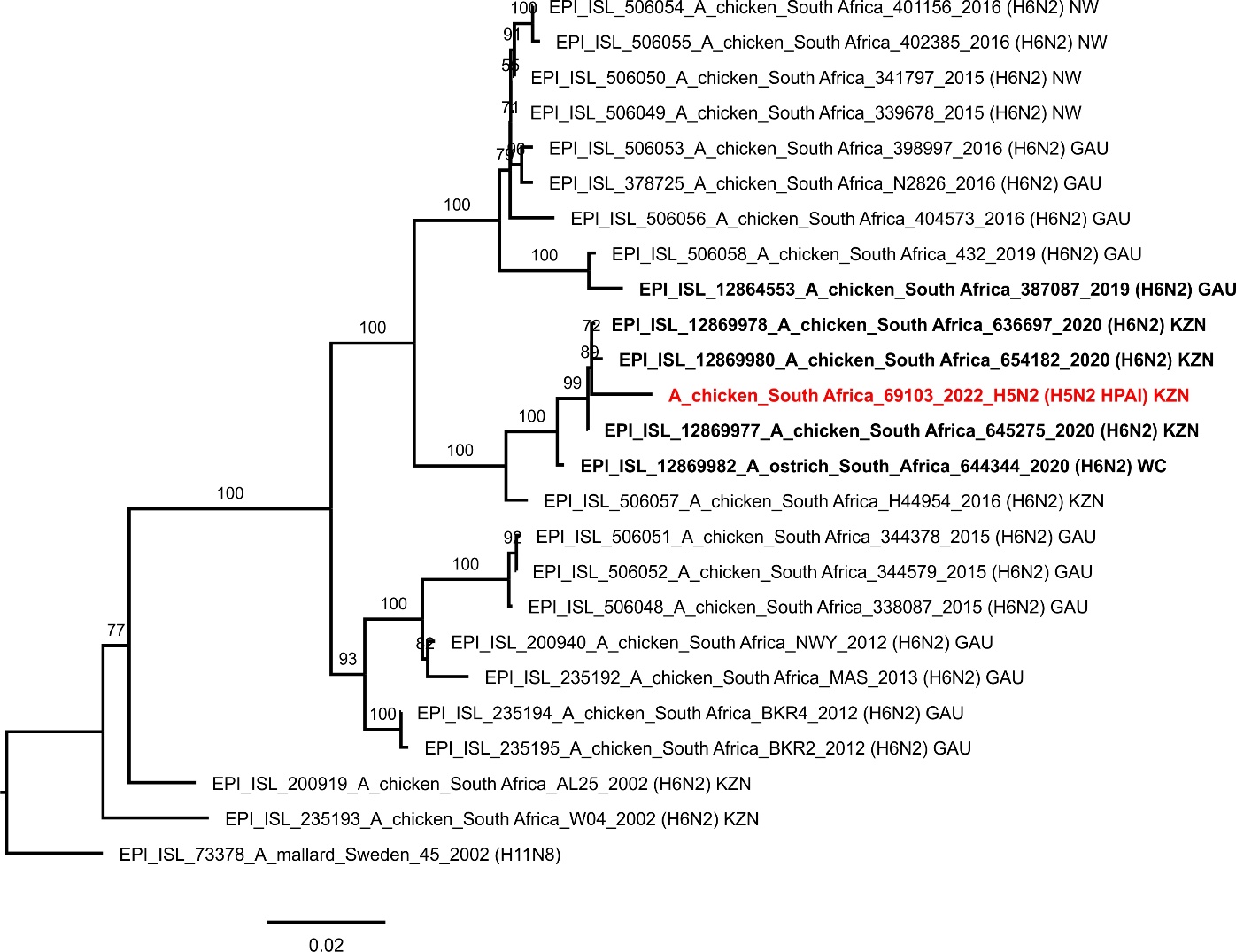


(i) PB2


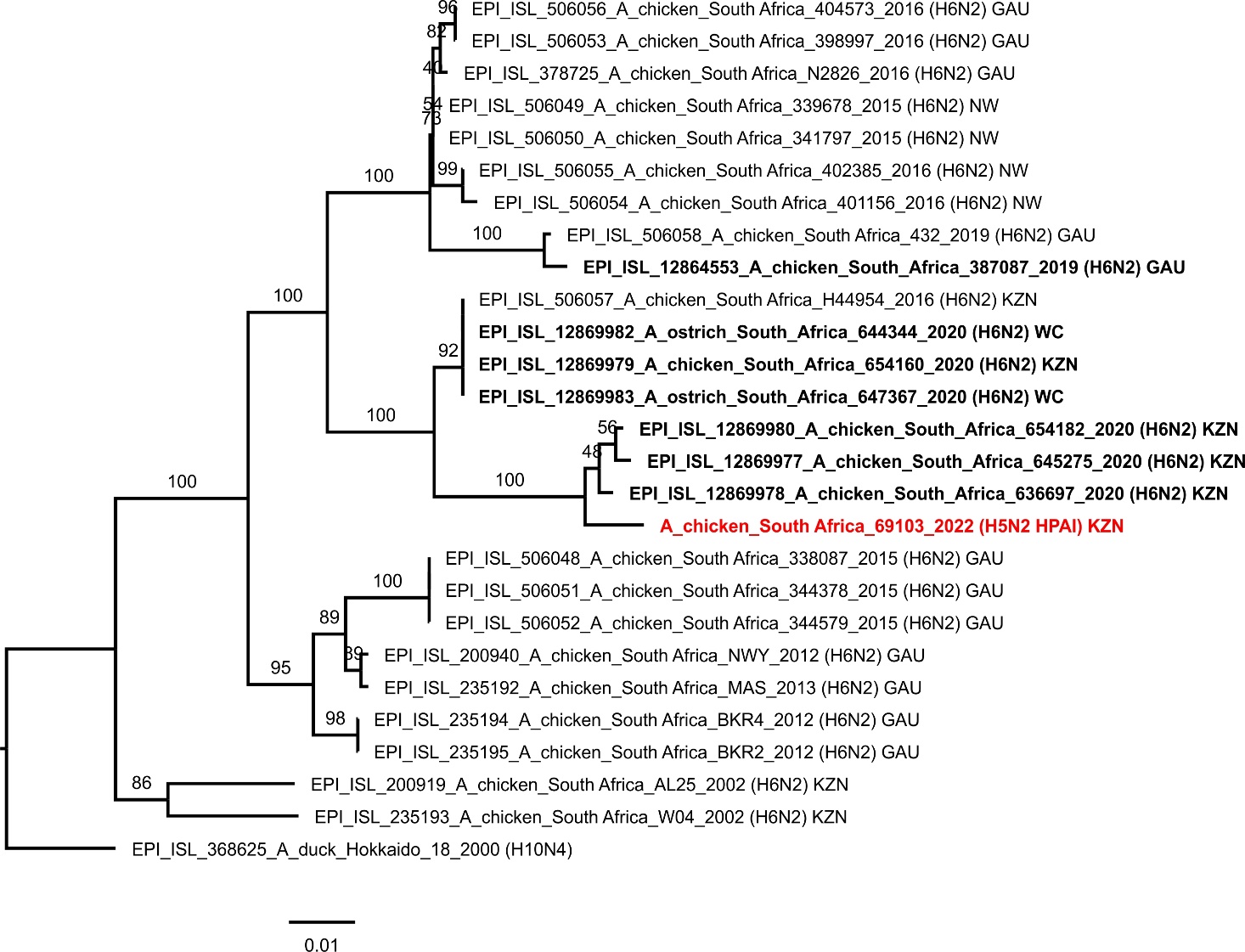


(ii) PB1


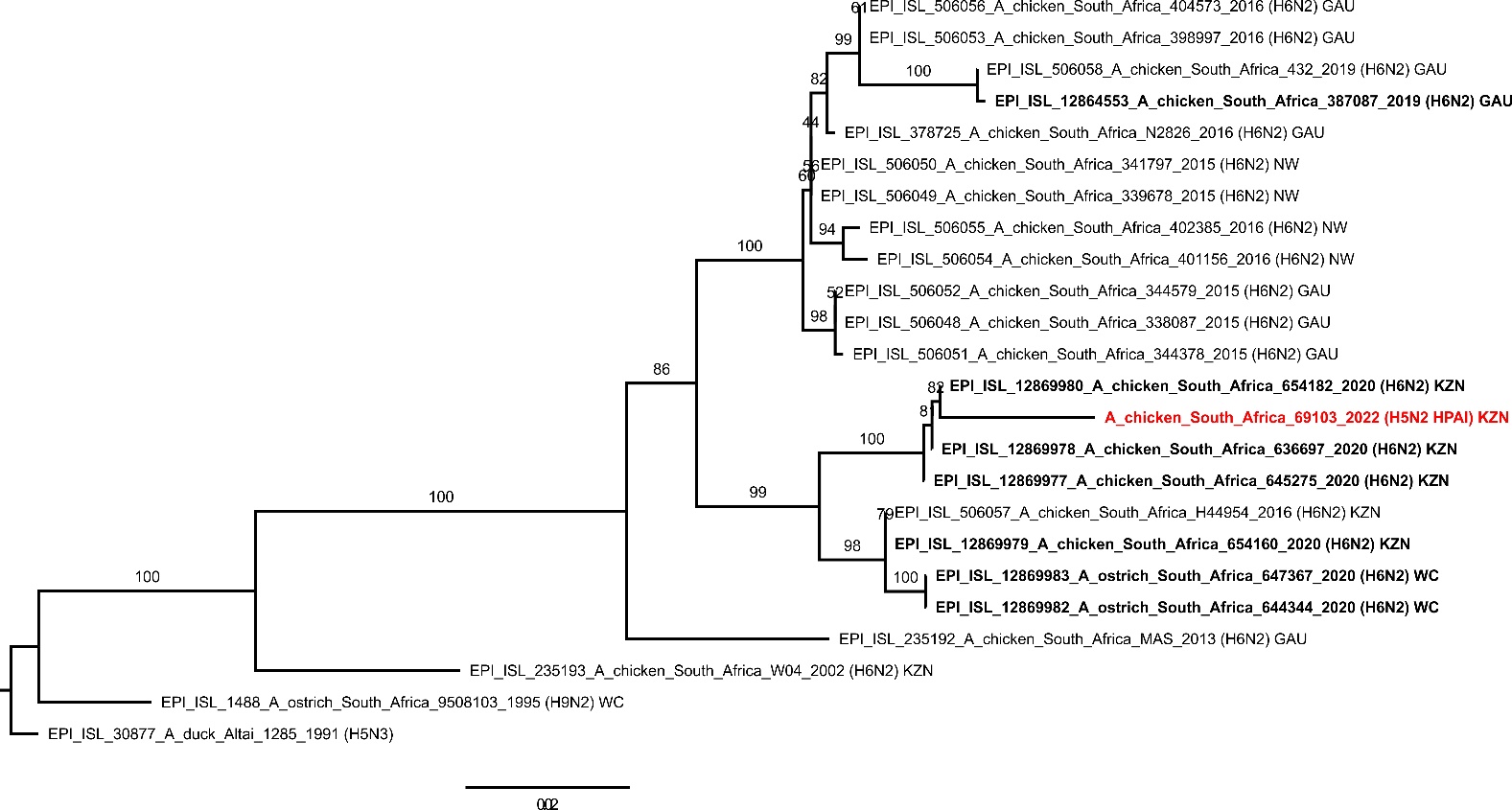


(iii) PA


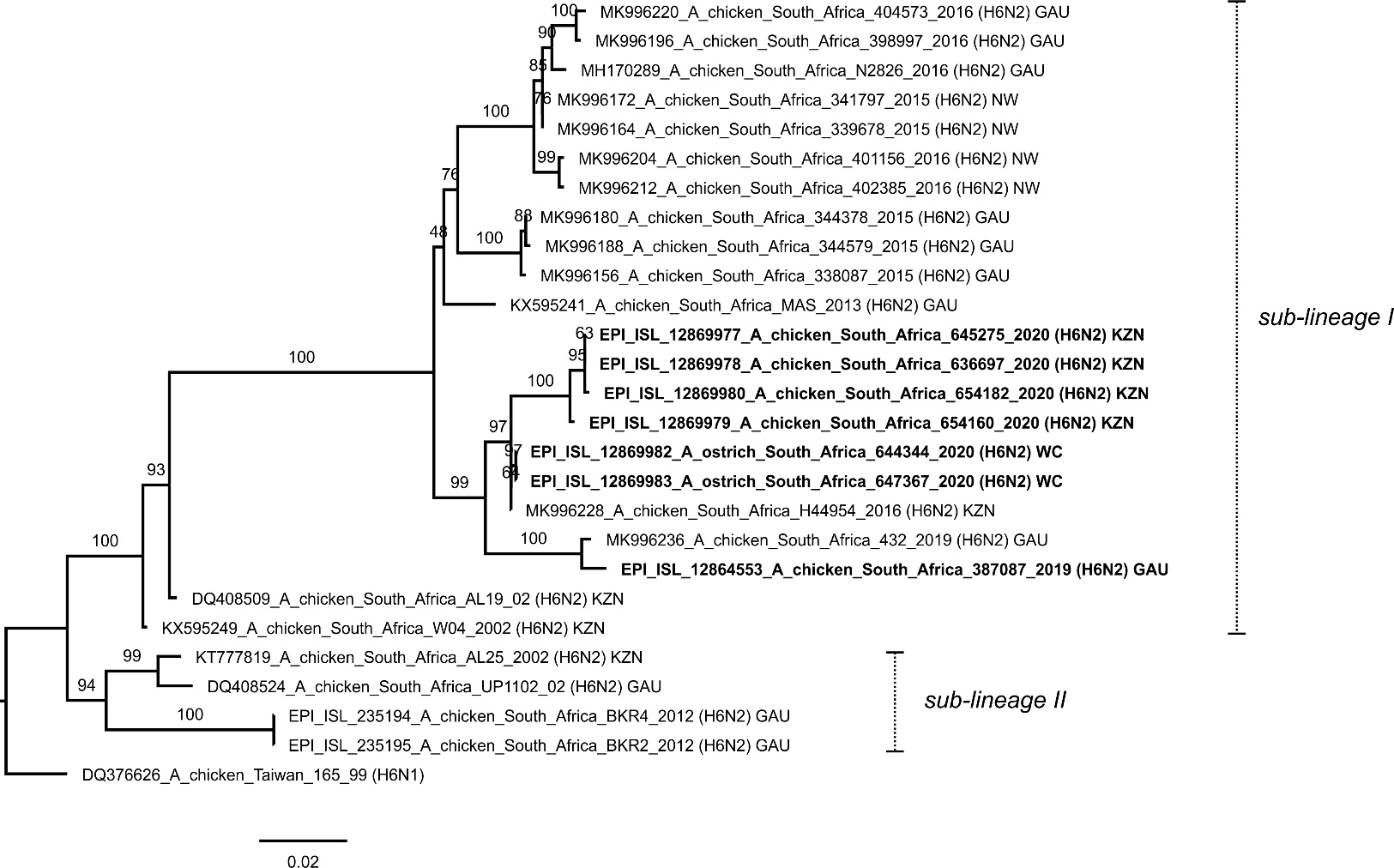


(iv) HA


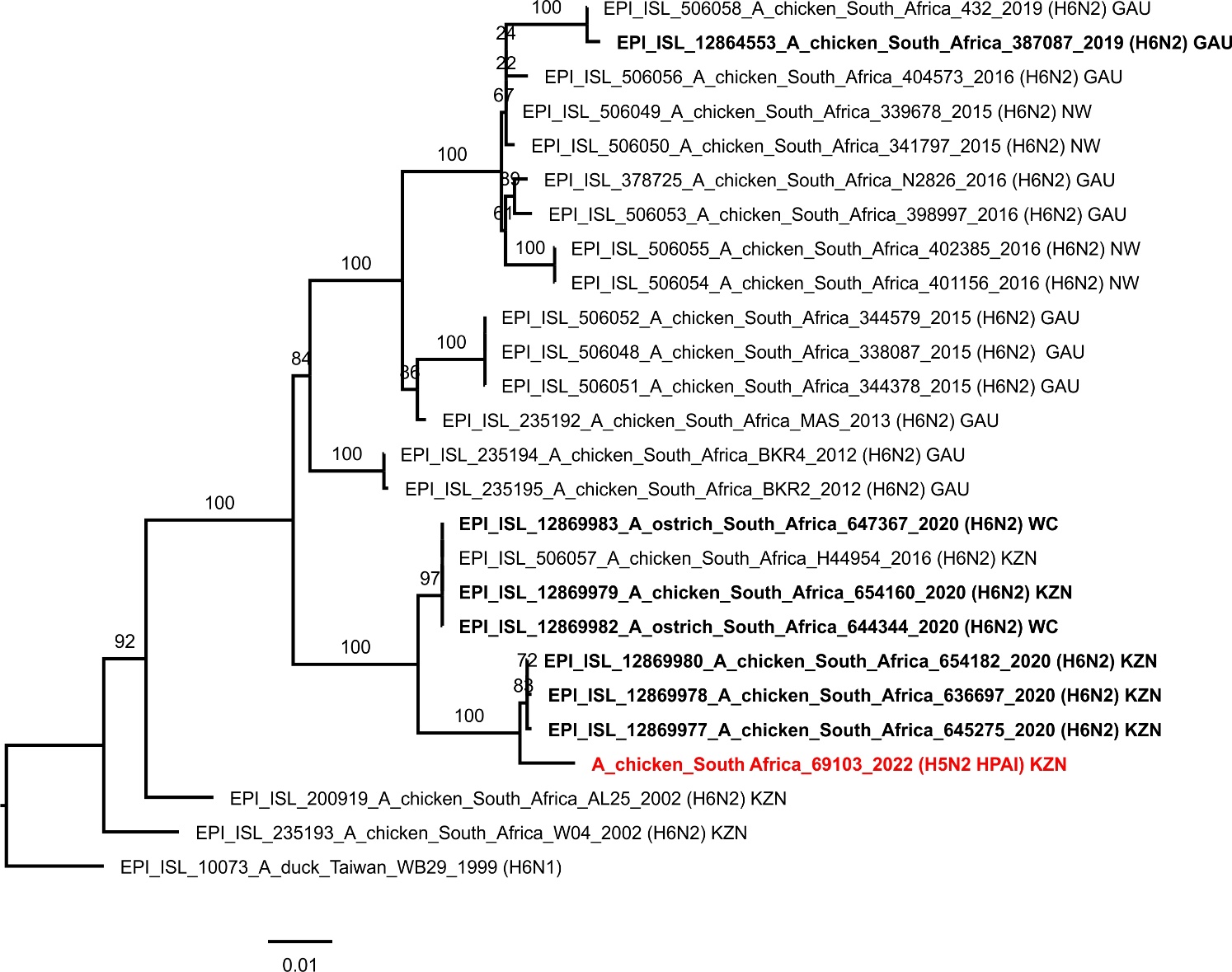


(v) NP


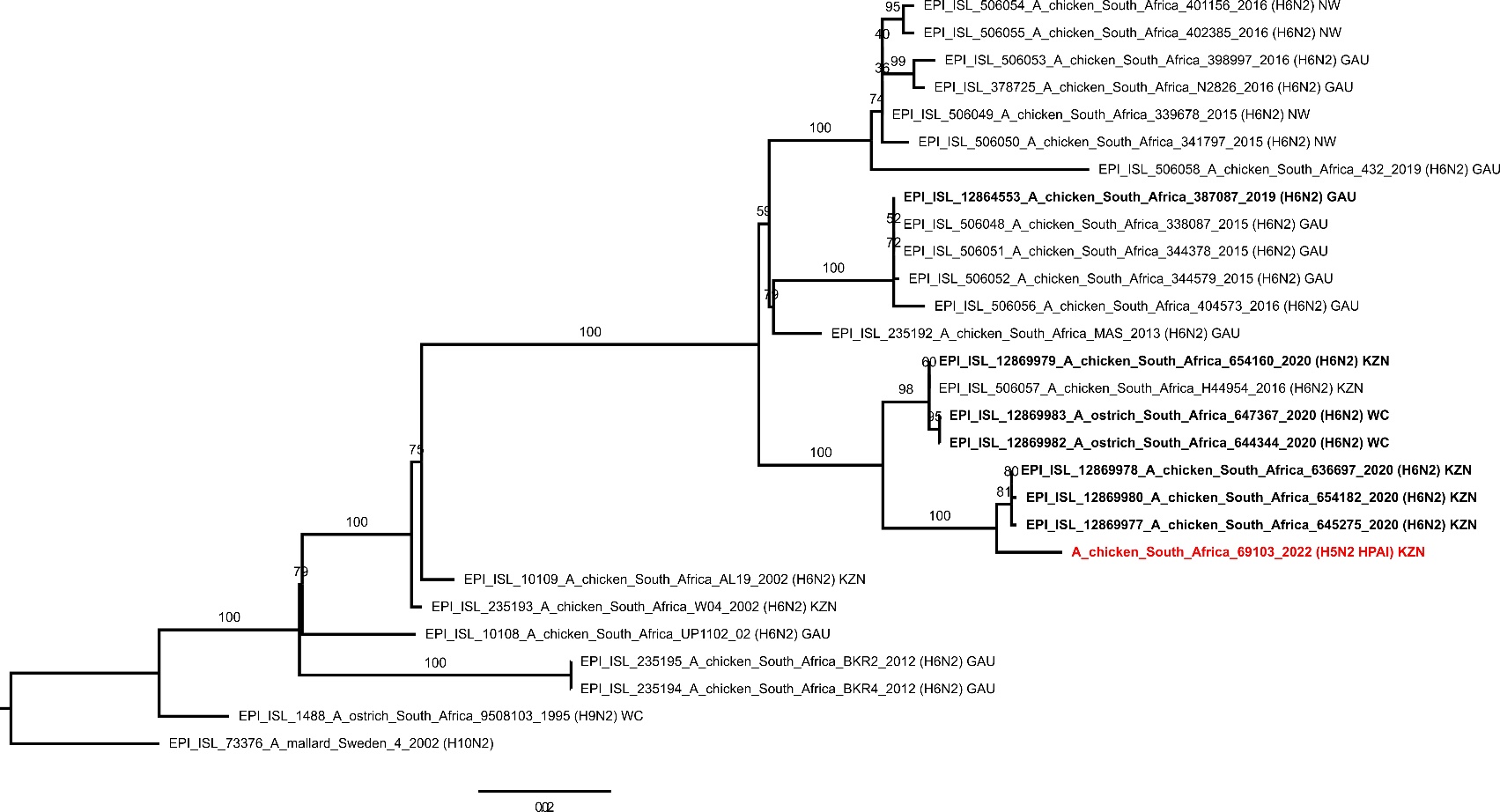


(vi) NA


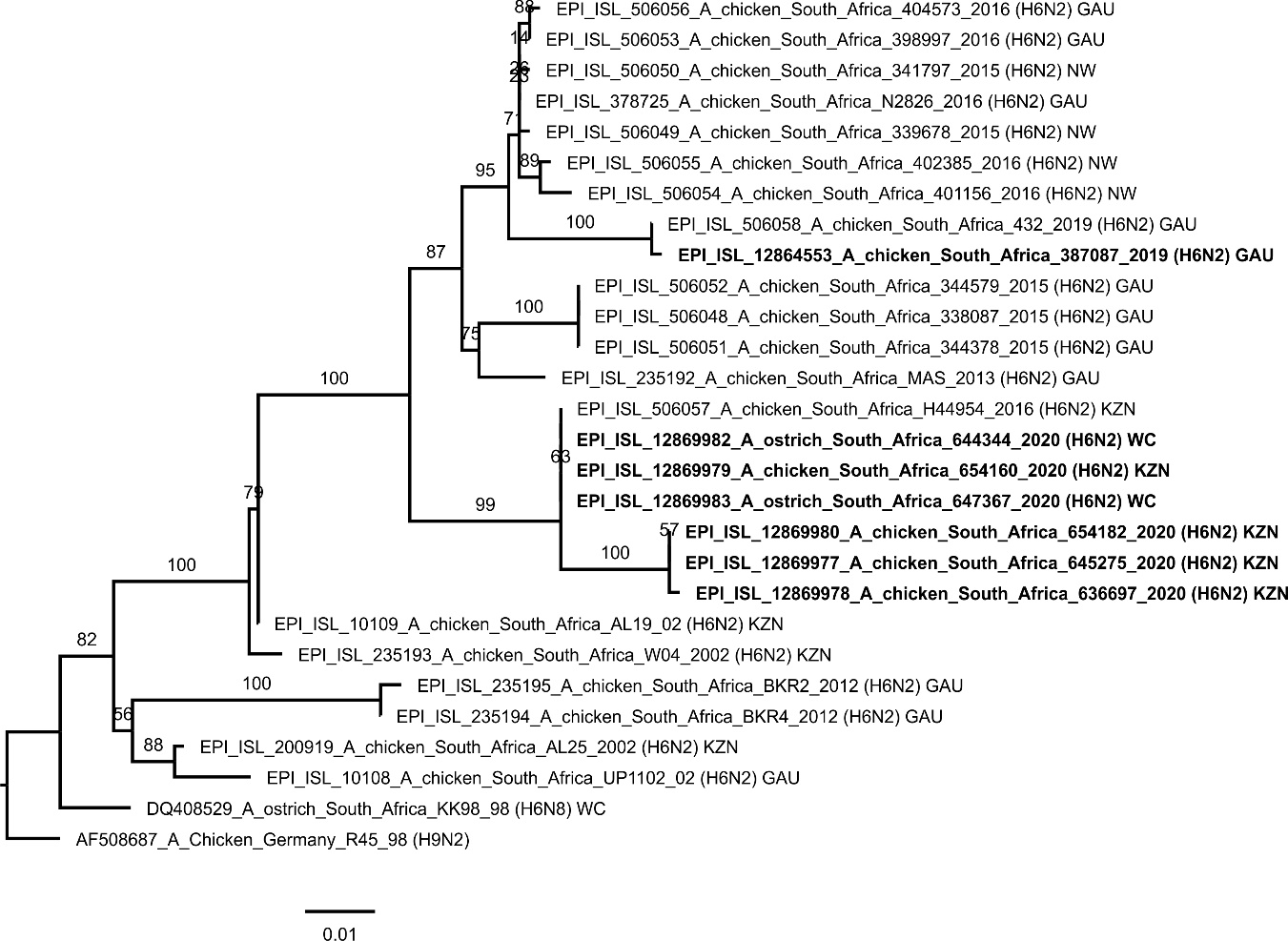


(vii) M


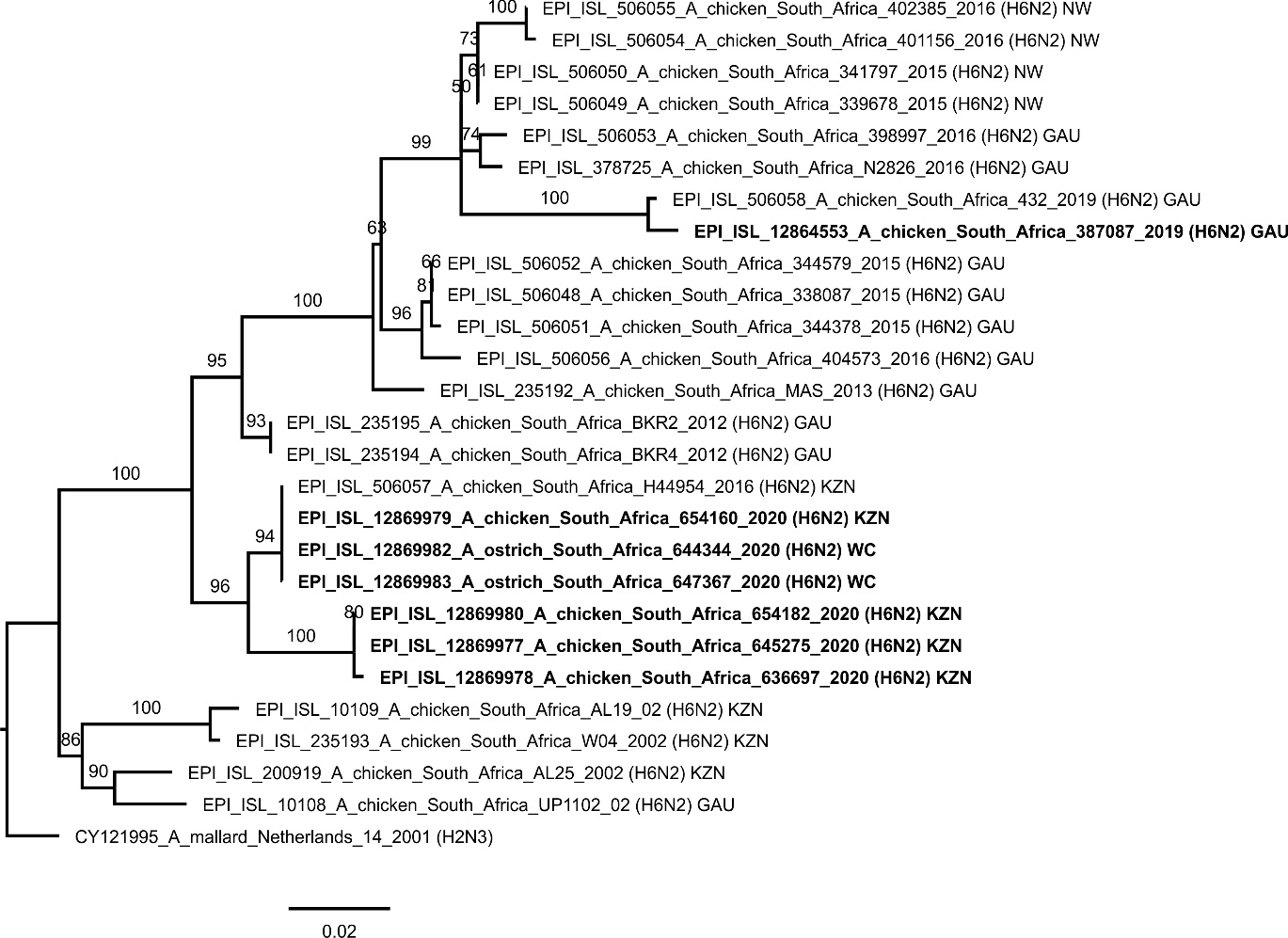


(viii) NS

**Supplemental Figure 1(a).** Maximum likelihood phylogenetic trees for the eight genome segments of South African H6N2 viruses and the H5N2 HPAI reassortant virus from KwaZulu-Natal province (in red). Viruses sequenced in this study are in boldface. A/chicken/South Africa/654160/2020 and A/ostrich/South Africa/647367/2020 are omitted from the PB2 tree due to their partial sequences. The provincial locations for each virus are abbreviated as follows: GAU- Gauteng; KZN- KwaZulu-Natal; NW- North-West; WC-Western Cape.


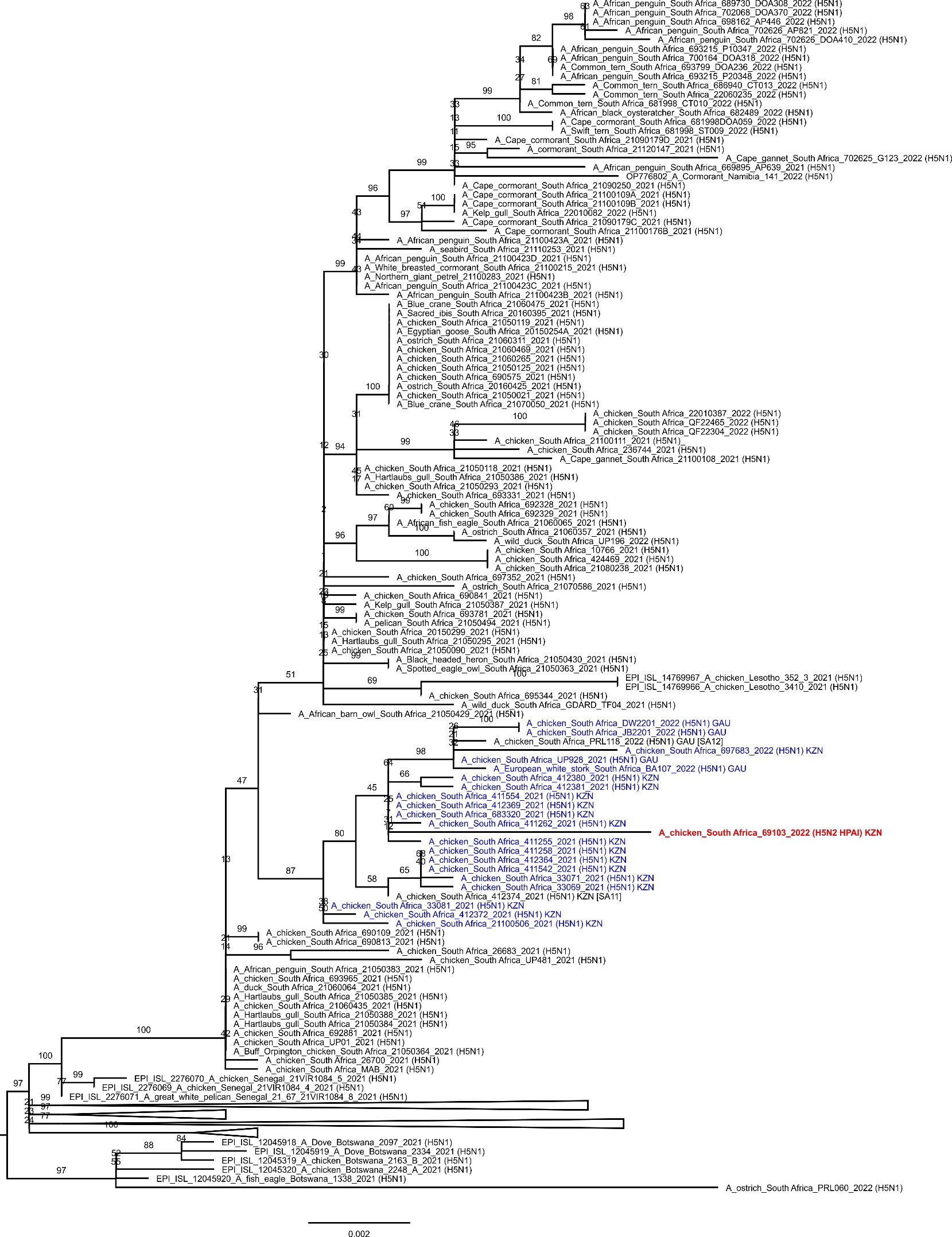


(i) HA


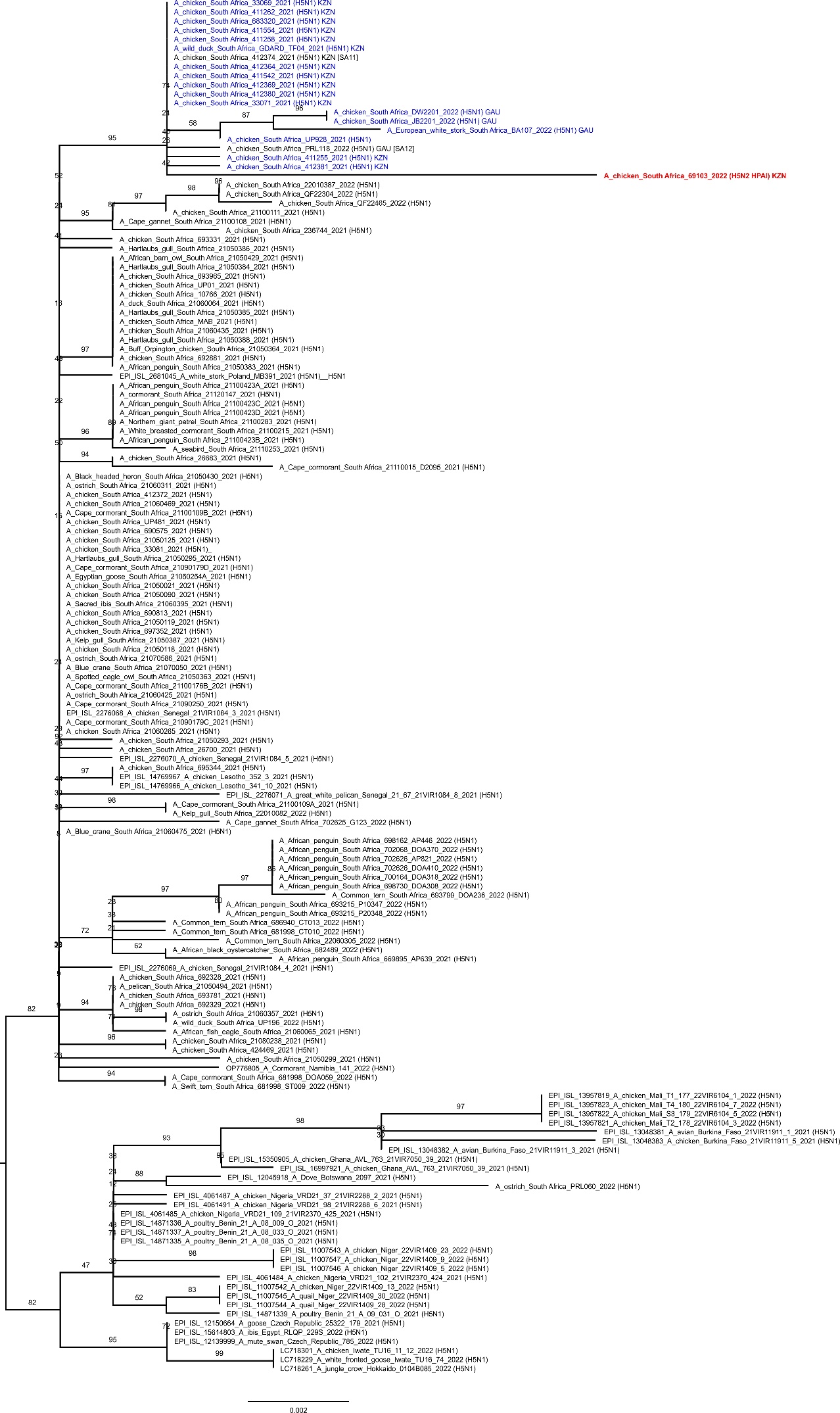


(ii) M


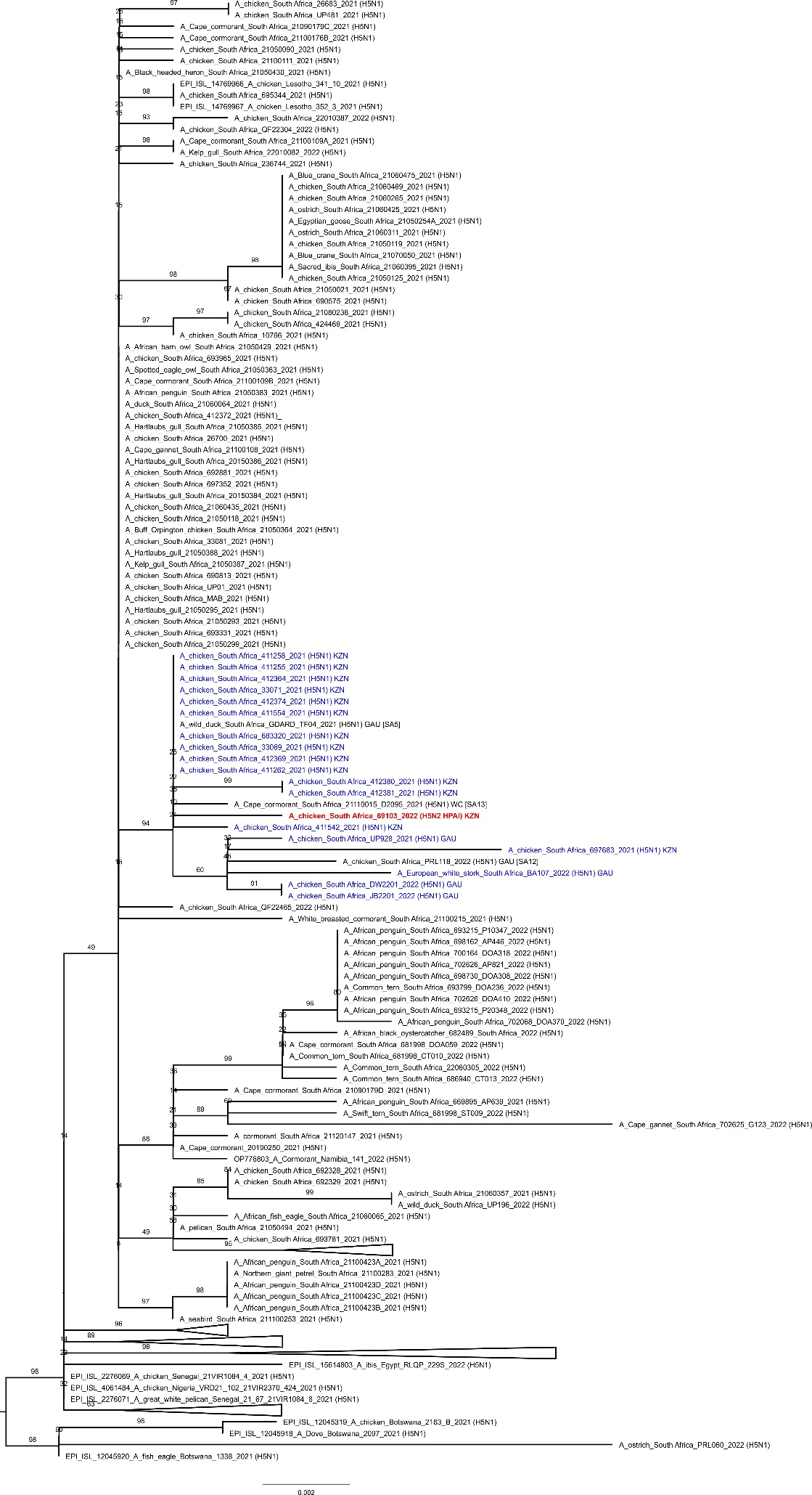


(iii) NS

**Supplemental Figure 1(b).** Maximum likelihood phylogenetic trees for the HA, M and NS genome segments of South African clade 2.3.4.4b H5N1 viruses and the H5N2 HPAI reassortant virus (in red). The members of sub-genotype SA10 are in blue. The provincial locations for the viruses in sub-genotype SA10 are abbreviated as follows: GAU- Gauteng; KZN- KwaZulu-Natal; WC-Western Cape.
